# A novel mitochondrial tRNA^Val^ T1658C mutation identified in a CPEO family

**Published:** 2010-08-25

**Authors:** Naihong Yan, Shuping Cai, Bo Guo, Yi Mou, Jing Zhu, Jun Chen, Ting Zhang, Ronghua Li, Xuyang Liu

**Affiliations:** 1 Ophthalmic Laboratories and Department of Ophthalmology, West China Hospital, Sichuan University, Chengdu, Sichuan, P.R. China; 2 Department of Medical Genetics, West China Hospital, Sichuan University, Chengdu, Sichuan, P.R. China

## Abstract

**Purpose:**

To analyze mitochondrial DNA (mt DNA) gene mutations in a 19-year-old female patient, who presented with chronic progressive external ophthalmoplegia (CPEO), together with her mother and younger sister.

**Methods:**

The diagnosis of mitochondrial myopathy was made based on clinical and biologic analysis. Histochemical methods were used to detect ragged-red fibers (RRFs) and ragged-blue fibers (RBFs) on a muscle biopsy of the patient. All mitochondrial gene DNA fragments of the patient, her mother, and younger sister were amplified by polymerase chain reaction. The products were sequenced and compared with reference databases.

**Results:**

A novel T1658C mutation and a known A10006G mutation were identified in the mitochondrial tRNA^Val^ gene and the tRNA^Gly^ gene, respectively, in the patient, her mother, and younger sister. The T1658C mutation changes the T loop structure of mitochondrial tRNA^Val^ and the A10006G mutation disturbs the D loop of mitochondrial tRNA^Gly^.

**Conclusions:**

The T1658C and A10006G mutations of mtDNA may be responsible for the pathogenesis of the patient with CPEO.

## Introduction

Mitochondrial myopathies, usually maternally inherited and involving multiple organs, are a group of neuromuscular diseases caused by mitochondrial dysfunction [1]. Most mitochondrial myopathies occur before the age of 20 and often begin with exercise intolerance or muscle weakness. Prognoses for the disorders are poor, with severity ranging from progressive weakness to death [2].

Chronic progressive external ophthalmoplegia (CPEO) is the most common mitochondrial myopathy, making up an estimated two-thirds of all incidences of mitochondrial-associated disorders. Patients typically present with progressive paralysis of the extraocular muscles. Patients usually experience bilateral, symmetric, and progressive ptosis, followed by ophthalmoparesis months or years later. Deletions and mutations of mitochondrial DNA lead to CPEO, and several point mutations in mitochondrial tRNA (mt tRNA) genes are known to be associated with CPEO [3].

## Methods

### Patient and controls

A 19-year-old female Han Chinese patient presented with progressive bilateral blepharoptosis, which was first noticed at age 13. The symptoms were neither alleviated in the morning nor aggravated in the evening. She was prone to fatigue after exercise. The family medical history was not significant. A complete clinical examination of both her mother and sister had been performed and no disorders were noticed. This patient and her family were recruited from the clinic at the Department of Ophthalmology, West China Hospital, Sichuan University, Chengdu, People’s Republic of China. One hundred and forty-eight control subjects, random normal Han Chinese living in the same region as the patient, were also recruited from the West China Hospital. The study was approved by the West China Hospital Sichuan University Institute Review Board. This study adhered to the tenets of the Declaration of Helsinki.

### Histological examination

An open muscle biopsy was performed by obtaining tissue through a small incision from the patient’s left musculus biceps brachii. The biopsy was divided into two parts: one part was snap frozen in isopentane pre-cooled in liquid nitrogen for cryosections, and the other part was fixed in 10% buffered formalin for routine paraffin embedded sections. The sections were stained with hematoxylin-eosin (H&E), modified Gomori trichrome (MGT), and succinate dehydrogenase (SDH).

### Mutation analysis of the mitochondrial genome

Genomic DNA was isolated from whole blood of the patient, her mother, and sister using the phenol-chloroform method. First, the three individuals’ DNA fragments spanning the whole mitochondrial gene were amplified by PCR using 24 pairs of oliodeoxynucleotides (Table 1) which sets the light (L) strand and the heavy (H) strand [4]. The overlapping PCR products were purified and subsequently analyzed by direct sequencing in an ABI 3700 automated DNA sequencer using the Big Dye Terminator Cycle sequencing reaction kit. Next, two mitochondrial regions covering 1658 and 10006 from 148 control DNA samples were amplified by PCR and sequenced.

**Table 1:** Primers used in polymerase chain reaction for amplification of the entire mitochondrial genome.

**Primer**	**Sequence (5’→3’)**	**Annealing temperature (°C)**	**Size of product**
1	Forward: CTCCTCAAAGCAATACACTG	56	839
	Reverse: TGCTAAATCCACCTTCGACC		
2	Forward: CGATCAACCTCACCACCTCT	57	801
	Reverse: TGGACAACCAGCTATCACCA		
3	Forward: GGACTAACCCCTATACCTTCTGC	58	859
	Reverse: GGCAGGTCAATTTCACTGGT		
4	Forward: AAATCTTACCCCGCCTGTTT	57	886
	Reverse: AGGAATGCCATTGCGATTAG		
5	Forward: TACTTCACAAAGCGCCTTCC	57	831
	Reverse: ATGAAGAATAGGGCGAAGGG		
6	Forward: TGGCTCCTTTAACCTCTCCA	56	903
	Reverse: AAGGATTATGGATGCGGTTG		
7	Forward: ACTAATTAATCCCCTGGCCC	57	978
	Reverse: AATGGGGTGGGTTTTGTATG		
8	Forward: CTAACCGGCTTTTTGCCC	56	813
	Reverse: ACCTAGAAGGTTGCCTGGCT		
9	Forward: GAGGCCTAACCCCTGTCTTT	59	827
	Reverse: ATTCCGAAGCCTGGTAGGAT		
10	Forward: CTCTTCGTCTGATCCGTCCT	58	885
	Reverse: AGCGAAGGCTTCTCAAATCA		
11	Forward: ACGCCAAAATCCATTTCACT	55	986
	Reverse: CGGGAATTGCATCTGTTTTT		
12	Forward: ACGAGTACACCGACTACGGC	58	909
	Reverse: TGGGTGGTTGGTGTAAATGA		
13	Forward: TTTCCCCCTCTATTGATCCC	57	815
	Reverse: GTGGCCTTGGTATGTGCTTT		
14	Forward: CCCACCAATCACATGCCTAT	57	939
	Reverse: TGTAGCCGTTGAGTTGTGGT		
15	Forward: TCTCCATCTATTGATGAGGGTCT	58	892
	Reverse: AATTAGGCTGTGGGTGGTTG		
16	Forward: GCCATACTAGTCTTTGCCGC	60	859
	Reverse: TTGAGAATGAGTGTGAGGCG		
17	Forward: TCACTCTCACTGCCCAAGAA	56	801
	Reverse: GGAGAATGGGGGATAGGTGT		
18	Forward: TATCACTCTCCTACTTACAG	55	865
	Reverse: AGAAGGATATAATTCCTACG		
19	Forward: AAACAACCCAGCTCTCCCTAA	56	976
	Reverse: CGATGATGTGGTCTTTGGA		
20	Forward: ACATCTGTACCCACGCCTTC	58	969
	Reverse: AGAGGGGTCAGGGTTGATTC		
21	Forward: GCATAATTAAACTTTACTTC	55	937
	Reverse: AGAATATTGAGGCGCCATTG		
22	Forward: TGAAACTTCGGCTCACTCCT	57	1161
	Reverse: AGCTTTGGGTGCTAATGGTG		
23	Forward: TCATTGGACAAGTAGCATCC	55	809
	Reverse: GAGTGGTTAATAGGGTGATAG		
24	Forward: CACCATCCTCCGTGAAATCA	55	963
	Reverse: AGGCTAAGCGTTTTGAGCTG		

Summary of the primer sequences, annealing temperatures, and size of products used for the amplification of the entire mitochondrial genome.

## Results

The patient—the only subject manifesting CPEO in the pedigree—had previously undergone thorough ophthalmic examinations. Her visual acuity was 20/80 in the right eye (O.D.) and 20/100 in the left eye (O.S.). Bilateral and asymmetric ptosis was noticed. The palpebral aperture was 3 mm for the O.D. and 5 mm for the O.S. The abduction and adduction of the left eye were mildly limited, yet the supraduction and deorsumduction appeared normal. The movement of the left eye was normal. Binocular diplopia was noticed in all directions. Both pupils were equal, round, and reactive to light. The anterior segment was unremarkable. Visual evoked potential showed P100 latency (positive peak at around 100 ms) was delayed in both eyes and P100 amplitude was reduced in the O.D.

**Figure 1:**
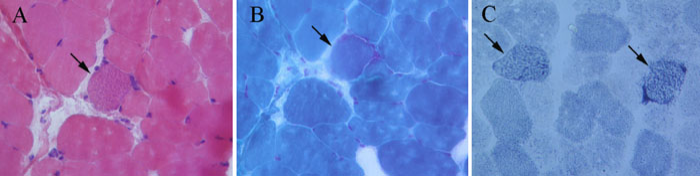
Histological examination of left biceps from the patient. **A**: H&E staining shows an RRF (arrow). **B**: MGT staining shows an atypical RRF (arrow). **C**: SDH staining shows ragged-blue fibers (arrows). Magnification 400×.

Other examinations included neurologic examinations, laboratory tests, and a skeletal muscle biopsy. The muscle strength of proximal extremities was normal (5/5), but was slightly weak at the distal end (4/5). The muscular tension of extremities was within normal limits and myotonia was absent. Deep reflexes and sensation were normal. Routine blood and urine tests were unremarkable. A magnetic resonance imaging (MRI) scan of the head showed no abnormality. The antinuclear antibody (ANA) was negative. An electromyologram (EMG) showed the right ulnar nerve has no abnormality in low and high frequency stimulation. Histological examinations showed ragged-red fibers (RRFs) and ragged-blue fibers (RBFs) on the patient’s muscle biopsy (Figure 1), consistent with the pathological features of mitochondrial myopathy.

**Figure 2:**
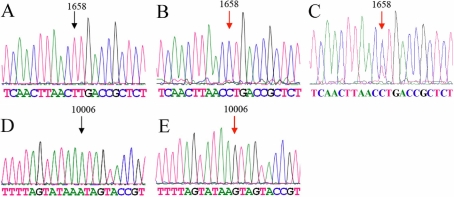
mt tRNA sequence results from the affected subject (Mutant) and the control (Wild type). **A**, **B**, and **C**: T1658C mutation (**A**: Wild type, **B**: Mutant, **C**: T/C Heteroplasmy). **D** and **E**: A10006G mutation (**D**: Wild type, **E**: Mutant). Arrows indicate the nucleotide changes.

**Figure 3:**
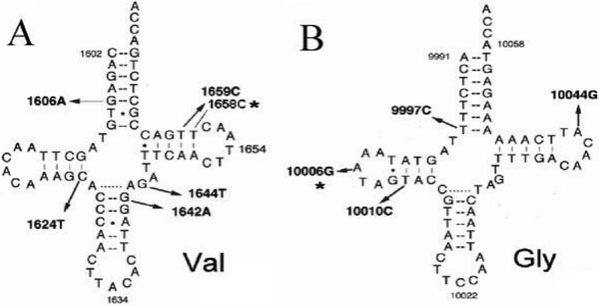
Mutation positions located at mt tRNAs in this patient. **A**: tRNA^Val^, **B**: tRNA^Gly^. *show the mutations in tRNA^Val^ (position 57) and tRNA^Gly^ (position 16).

To determine the role of mitochondrial abnormalities in the pathogenesis of this patient with CPEO, the 24 PCR fragments spanning the entire mitochondrial genome of the affected individual, her mother, and younger sister were analyzed by direct sequencing. The comparison of the resultant sequences with the Revised Cambridge Reference Sequence [5] revealed several nucleotide changes, as shown in Table 2, Figure 2, and Figure 3. The patient’s mitochondrial sequence results are identical to those of her mother and sister, except the T1658C mutation of the patient’s young sister is T/C heterozygous in the mitochondrial tRNA^Val^ gene. There were no T1658C or A10006G mutations in the148 control subjects.

**Table 2:** mtDNA nucleotide changes in the affected subject.

**Gene**	**Position**	**Nucleotide change**	**Amino acid change**	Previously reported*
D-loop	73	A→G		+
	263	A→G		+
	310	T→CTC		+
	489	T→C		+
	514	C→DEL		+
	515	A→DEL		+
	568	C→CCCC		+
	16223	C→T		+
	16362	T→C		+
	16519	T→C		+
12SrNA	750	A→G		+
	1382	A→C		+
	1438	A→G		+
**TV**	**1658**	**T→C**		**-**
16SrNA	2706	A→G		+
	3010	G→A		+
ND1	4769	A→G		+
ND2	4883	C→T		+
	5178	C→A	L→M	+
CO1	7028	C→T		+
CO2	8020	G→A		+
ATP8	8414	C→T	L→F	+
ATP6	8701	A→G	T→A	+
	8860	A→G		+
	8964	C→T		+
CO3	9296	C→T		+
	9540	T→C		+
	9545	A→G		+
	9824	T→A		+
**TG**	**10006**	**A→G**		**+**
ND3	10398	A→G	T→A	+
	10400	C→T		+
ND4	10790	T→C	SYN	-
	10873	T→C		+
	11014	C→T	SYN	-
	11719	G→A		+
ND5	12705	C→T		+
	13866	A→G	SYN	-
ND6	14668	C→T		+
CYB	14766	C→T	T→I	+
	14783	T→C		+
	15043	G→A		+
	15301	G→A		+
	15326	A→G	T→A	+

*See http://www.mitomap.org. The boldfaced are pathogenic mutations.

## Discussion

Chronic progressive external ophthalmoplegia, characterized by limited ocular motility in all directions of gaze and ptosis, is the most common manifestation of mitochondrial myopathy and usually occurs in young adulthood. Ptosis is usually the first clinical sign, but ophthalmoplegia may not become apparent for months or even years. The ptosis is usually bilateral and symmetric.

The diagnosis of CPEO relies upon a combination of different approaches, including clinical data, neurologic examinations, laboratory tests, and a skeletal muscle biopsy. Ragged-red fibers on muscle biopsies are observed in a wide variety of mitochondrial diseases. The presence of more than 2% RRFs on skeletal muscle biopsies can be considered one of the criteria required for the diagnosis of mitochondrial disease. Figure 1 shows RRFs and RBFs on the patient’s muscle biopsy. Stained with MGT staining, one can see an accumulation of enlarged mitochondria in the RRFs. Therefore, changes and dysfunction of mitochondria are the pathogenesis of CPEO.

Over the past two decades, numerous genetic causes of CPEO have been described [6-30]. Most present at mt tRNA genes, affecting mitochondrial tRNA^Leu(UUR)^, tRNA^Ile^, tRNA^Ala^, tRNA^Asn^, tRNA^Lys^, tRNA^Leu(CUN)^, and tRNA^Gly^ genes, including A3243G, T3250C, and C3254T in the tRNA^Leu(UUR)^ gene; T4274C, T4285C, G4298A, and G4309A in the tRNA^Ile^ gene; T5628C in the tRNA^Ala^ gene; T5692C and G5703A in the tRNA^Asn^ gene; G12294A, A12308G, T12311C, and 12315A in the tRNA^Leu(CUN)^ gene; and an A10006G mutation in the tRNA^Gly^ genes [6-30]. Most of these point mutations in mt tRNA genes have been proved only once and appeared to be limited to one patient or one family; some point mutations have been reported in different pedigrees [22]. Therefore, mt tRNA mutations play a pivotal role in the pathogenesis of CPEO.

There are 44 nucleotide changes in this CPEO subject’s mitochondrial genome that belong to haplogroup D4f. Most nucleotide changes are polymorphisms and are not associated with human diseases. However, two mutations in this subject, T1658C in the tRNA^Val^ gene and A10006G in the tRNA^Gly^ gene, may be responsible for this disorder.

In fact, Lauber et al. [22] first reported a CPEO patient with a tRNA^Gly^ A10006G mutation. Sternberg et al. [16] also found this mutation in a patient with oculomotor myopathy. Münscher et al. [14] identified tRNA^Gly^ A10006G, a mutation known to be associated with aging, in patients with chronic intestinal pseudo-obstruction (CIPO) and CPEO. Our results, as exhibited in Figure 2D,E and Figure 3B, show the A10006G mutation is located at position 16 on the D loop of tRNA^Gly^. Most importantly, this mutation changes the structure of the D loop of mt tRNA^Gly^. T1658C is a novel mutation, located at position 57 of tRNA^Val^. Figure 2A-C show that wild type T at position 57 matches with A at position 50 in tRNA^Val^. When position 57 T changes to C, the mutant 57 C does not match with position 50 A. Therefore, one base pair on the T arm of tRNA^Val^ is split and the T loop is extended.

Mitochondrion has its own tRNAs to carry amino acids to synthesize mitochondrial proteins essential for ATP production. Each tRNA is charged with the proper amino acid via a covalent ester bond at their 3′ end by the specific aminoacyl-tRNA synthetase. In our study, the structural changes of mt tRNA^Gly^ and tRNA^Val^, like the A3243G mutation in mt tRNA^Leu(UUR)^ [30,31], may influence their structural stability, modifications, 3′ end processing, and aminoacylations and decrease mitochondrial protein synthesis and ATP production. Valine is one of these essential amino acids. The defective mt tRNA^Val^ will reduce the transportation efficiency for valine, leading to insufficient valine in the mitochondrial translation. Therefore, the T1658C mutation leads to the T loop structural change of mt tRNA^Val^, indicating a role in the development of CPEO in this individual. Furthermore, the mitochondrial sequence of the patient’s younger sister is 1658 T/C heterozygous; however, her sister has not manifested CPEO. 

Muscle, especially extraocular muscle, consumes a lot of energy. Mitochondrial tRNA mutations may cause the dysfunction of mitochondrial translation. Our results suggest that T1658C, a newly identified mutation as an independent factor or one associated with the A10006G mutation, may contribute to the pathogenesis of CPEO. However, the molecular mechanisms of T1658C that cause CPEO and the confounding effects of T1658C and A10006G mutations have yet to be elucidated. To determine the role of the mutations (T1658C and /or A10006G) in mitochondrial dysfunction, the lymphoblastoid cell lines should be generated—using blood from the patient and her family members, as well as control subjects—and studied. Further study of the pathogenetic role of mtT1658C in CPEO patients with this mitochondrial mutation only is also needed.

It is noted that the DNA extracted from muscle tissue should be more appropriate for use in the mutational study than that extracted from blood since the changes in muscular mtDNA would be more convincing in explaining the involvement of selective extraocular muscles in CPEO.
